# Citrate and Polyvinylpyrrolidone Stabilized Silver Nanoparticles as Selective Colorimetric Sensor for Aluminum (III) Ions in Real Water Samples

**DOI:** 10.3390/ma13061373

**Published:** 2020-03-18

**Authors:** Paula Ruíz del Portal-Vázquez, Germán López-Pérez, Rafael Prado-Gotor, Cristina Román-Hidalgo, María Jesús Martín-Valero

**Affiliations:** 1 Department of Analytical Chemistry, Faculty of Chemistry, University of Sevilla, 41012 Sevilla, Spain; pauruidel@gmail.com (P.R.d.P.-V.); croman2@us.es (C.R.-H.); 2 Department of Physical Chemistry, Faculty of Chemistry, University of Sevilla, 41012 Sevilla, Spain; gerlopez@us.es (G.L.-P.); pradogotor@us.es (R.P.-G.)

**Keywords:** silver nanoparticles, polyvinylpyrrolidone, Surface Plasmon Band deconvolution, sensoring, aluminum

## Abstract

The use of silver nanoparticles stabilized with citrate and polyvinylpyrrolidone as a sensor for aluminum ions determination is proposed in this paper. These non-functionalized and specific nanoparticles provide a highly selective and sensitive detection system for aluminum in acidic solutions. The synthesized nanoparticles were characterized by transmission electron microscopy. Surface plasmon band deconvolution analysis was applied to study the interaction between silver nanoparticles and aluminum ions in solution. The interaction band in the UV-visible region was used as an analytical signal for quantitation purposes. The proposed detection system offers an effective AND wide linearity range (0.1–10^3^ nM), specificity for Al(III) in THE presence of other metallic ions in solution, as well as high sensitivity (limit of detection = 40.5 nM). The proposed silver-nanoparticles-based sensor WAS successfully used for detecting Al(III) in real water samples.

## 1. Introduction

Aluminum is the most abundant metal in the earth’s crust and it is widely distributed. This aluminum IS combined with other elements, most commonly with oxygen, silicon, and fluorine. These chemical compounds are commonly found in soil, minerals (sapphires, rubies, turquoise), rocks (especially igneous rocks), and clays. Aluminum metal is used as a structural material in the construction, automotive, and aircraft industries; in the production of metal alloys; in the electric industry; in cooking utensils; as well as in food packaging and additives. Prolonged use of aluminum foil and vessels can increase the amount of aluminum in food [1,2,3]. Aluminum absorption in human intestines and posterior transportation to bones make this element potentially toxic due to its accumulation [4]. The relationship between aluminum deposition in bones and neurotoxicity has been established, including with diseases affecting the nervous system of the human body like Parkinson’s and Alzheimer’s [5,6,7,8]. The use of aluminum salts as coagulants in water treatment may increase concentrations of aluminum in finished water (tap or drinking water). Where residual concentrations are high, aluminum may be deposited in the distribution system. Disturbance of the deposits by changes in flow rate may increase aluminum levels at the tap and lead to undesirable color and turbidity [9,10].

In addition to humans, aluminum toxicity in water is dangerous to fish, and soil aluminum contamination affects agricultural production. Water and acidic soil are the two main sources of aluminum ions through which Al(III) enters the human food chain; plant roots are also adversely affected by the release of Al(III) ion from the acidic soil [11,12,13].

Considering the strong impact of aluminum on human health and the environment, Al(III) detection is important. Analytical techniques commonly used for detecting metal ions include ion selective electrodes [14], electrochemical methods [15], atomic absorption and emission spectrometry [16,17], and colorimetric sensors [18,19].

Metallic nanoparticles were proposed as colorimetric sensors for numerous metal ions in different kind of samples. Gold nanoparticles (AuNPs) as well as silver nanoparticles (AgNPs) have been used for these purposes [20,21,22,23,24,25,26]. Usually, AuNPs and AgNPs are functionalized to obtain specific and selective sensors for detecting the desired metallic ion. Chen et al. used triazole–ether functionalized gold nanoparticles for the colorimetric detection of Al(III) ions [27]. Citrate-capped gold nanoparticles were used for rapid visual detection of aluminum at concentrations down to 1.0 μM [18]. Ascorbic-acid-capped gold nanoparticles were used for the selective colorimetric detection of aluminum ions in ground water [28]. Liu et al. proposed a colorimetric assay for Al(III) based on alizarin red S-functionalized silver nanoparticles [4]. Silver nanoparticles stabilized by reduced glutathione (GSH-AgNPs) in the presence of L-cysteine were used for colorimetric detection of aluminum by Yang et al. [29]. Even stable silver nanoclusters have been used for the detection of aluminum ions [30]. Silver nanoparticles coated with citrate or polyvinylpyrrolidone (PVP) were demonstrated to be versatile due to their different applications in a wide variety of fields like electronic applications or cell imaging between others [31,32,33].

As reported in the literature, the use of metallic nanoparticles as a detection system for metal ions is based on the shift to longer wavelengths of the surface plasmon resonance (SPR) absorption band due to the presence of metallic ions. However, deconvolution analysis can be a powerful tool for characterizing the interaction band between metallic nanoparticles and molecules in solution. Prado-Gotor et al. studied the aggregation of chitosan and gold nanoparticles applying a deconvolution process, providing a useful tool for studying electrostatic and covalent linkages [34,35].

In this paper, a selective sensor for the detection of aluminum is proposed using silver nanoparticles stabilized with citrate ions and PVP. One of the main novelties of this research is the use of a surface plasmon band (SPB) deconvolution procedure as mathematical tool for isolating the aluminum–AgNPs interaction band, which is used as an analytical signal for aluminum quantitation procedures. Analytical quality parameters such as reproducibility, repeatability, precision, and sensitivity were calculated to validate the proposed sensor as an analytical method for aluminum determination. Finally, the method was successfully applied to the aluminum quantitation in different kinds of real water samples.

## 2. Materials and Methods

### 2.1. Chemicals 

Sodium citrate, cobalt nitrate, chromium nitrate, nickel nitrate, aluminum nitrate, copper nitrate, and iron nitrate were obtained from Panreac (Barcelona, Spain); silver nitrate, sodium borohydride, and acetonitrile were obtained from Merck (Darmstadt, Germany); tetrachloroauric acid, polyvinylpyrrolidone, and ethylene glycol were purchased to Sigma-Aldrich (Madrid, Spain); and nitric acid (68%) and ascorbic acid were obtained from VWR (Lovaina, Belgium). All reagents were of analytical grade. Solutions were prepared using deionized water (18 MΩ, Milli-Q, Millipore, Bedford, MA, USA). 

### 2.2. Apparatus

The working pH was adjusted using a pH meter GLP 21 Crison (Barcelona, Spain). A UNIVERSAL 320 centrifuge (Hettich-Tuttlingen, Germany) was used in the synthesis of silver nanoparticles stabilized with PVP. Absorption spectra of AgNPs were obtained with a Thermo Spectronic UNICAM UV 500 spectrophotometer (Loughborough, UK) equipped with plastic disposable cells with optic path of 10 mm and spectral resolution of 1 nm in the range of 220–800 nm. Transmission electron microscopy (TEM) measurements were recorded with a Phillips CM 200 electron microscope (Amsterdam, the Netherlands) working at 200 KV. An Inductive Coupled Plasma Mass Spectrometer (ICP-MS) (Agilent 8800 ICP-QQQ, Agilent Technologies, Santa Clara, CA, USA) was used for aluminum content measurements in water samples.

### 2.3. Silver Nanoparticles Synthesis

Different kinds of spherical AgNPs were synthesized that were stabilized with sodium citrate, sodium borohydride, and polyvinylpyrrolidone (PVP).

#### 2.3.1. Synthesis of PVP-AgNPs

Spherical AgNPs using PVP as polymer stabilizer were synthesized according to the method proposed by Wang et al. [36]. We added 10 mL of ethylene glycol to a mixture of 85 mg of AgNO_3_ (5 × 10^−5^ mol) and 83 mg of PVP (2.08 × 10^−6^ mol). The resulting solution was heated at 185 °C for 20 min. The resulting particles were separated by centrifugation at 300 rpm during 5 min. The obtained solid was washed several times with deionized water without further purification. The washed particles were added to 19.4 mL of a deionized water with 0.6 mL of a NaBH_4_ solution (10 mM). The obtained AgNPs were called PVP-AgNPs. 

#### 2.3.2. Synthesis of NaBH_4_-AgNPs

Spherical AgNPs were synthesized using NaBH_4_ as the reducing agent and the stabilizing agent, according to Van Dong et al. [37]. An aqueous solution (50 mL) of NaBH_4_ (2 mM) was placed in an ice water cooling bath for 20 min under stirring. Once the temperature of the solution decreased to 5 °C, 2 mL of AgNO_3_ (1 mM) was added into the NaBH_4_ solution at an approximate rate of one drop per second. The obtained AgNPs were called NaBH_4_-AgNPs.

#### 2.3.3. Synthesis of Citrate-AgNPs

Spherical AgNPs were synthesized using sodium citrate as the reducing agent and the stabilizing agent, according to Fang et al. [38]. We heated 50 mL of AgNO_3_ (1mM) to boil and 5 mL of 1% (w/v) sodium citrate was added drop by drop. The resulting solution was maintained at a boil with continuous stirring until the reaction was completed. The reaction finished when a consistent light yellow color appeared in solution. At this point, the solution was cooled to room temperature. The obtained AgNPs were called citrate-AgNPs. 

#### 2.3.4. Synthesis of Citrate+PVP-AgNPs

Spherical AgNPs were synthesized by using aqueous solutions of NaBH_4_ acting as he reducing agent, and sodium citrate and PVP as the stabilizing agents [37]. We added 0.5 mL of sodium citrate (30 mM) into a flask, diluting with 50 mL of deionized water. After, 1 mL of AgNO_3_ (5 mM) and 0.5 mL of a NaBH_4_ (50 mM), freshly prepared, were quickly added. The obtained suspension immediately turned a light yellow color. At this point, 0.5 mL of PVP (5 mg/mL) was added and after 30 min the reaction was completed, which was identified by the change to a darker yellow color on the solution. The obtained AgNPs were called citrate+PVP-AgNPs.

All the synthesized silver nanoparticles solutions were stored under light preservation and refrigerated at 4 °C.

## 3. Results and Discussion

### 3.1. AgNPs Characterization

The four types of synthesized AgNPs were characterized using different spectroscopic techniques. Molecular absorption spectroscopy in the UV-visible range (300–800 nm) was used for studying the optical absorption features. PVP-AgNPs and citrate-AgNPs spectra showed an absorption maximum at a wavelength of 424 nm. For the spectra corresponding to NaBH_4_-AgNPs and citrate+PVP-AgNPs, the maximums of absorption appeared at around 400 nm (392 and 397 nm, respectively). 

**Figure 1:**
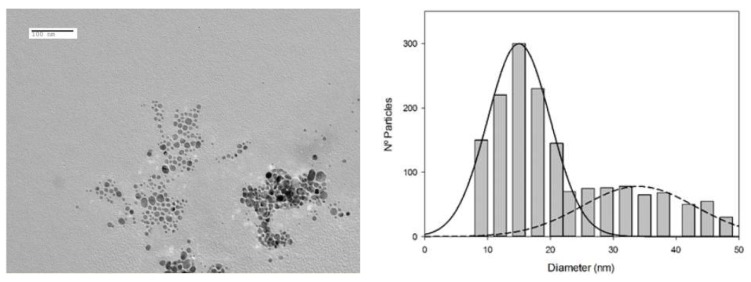
TEM image showing the shape and size of the citrate+PVP-AgNPs and calculated histogram to determine their average size from the TEM images.

TEM was used to obtain information about the size and morphology of the synthesized nanoparticles. The ImageJ software package (ver 1.52k29, National Institute of Health, Bethesda, MD, USA) was used to calculate the nanoparticles size from the corresponding micrographs. Table 1 summarizes the most important features of the synthesized nanoparticles. In all cases, a spherical morphology was observed, with the nanoparticles sizes varying depending on the capping agent. A smaller size corresponded to the NaBH_4_-AgNPs, whereas PVP-AgNPs showed an intermediate size. Citrate-AgNPs and Citrate+PVP-AgNPs showed a certain degree of polydispersity but, in all cases, the smaller sized nanoparticles were more abundant. As a representative example, Figure 1 shows the TEM image and the calculated histogram of the citrate+PVP-AgNPs. Two different populations of nanoparticles can be observed. The most abundant nanoparticles are between 10 and 20 nm in size, though bigger nanoparticles with a diameter of 25 to 45 nm were also found in a minor proportion. The maximum absorption wavelength ranged from 392 to 424 nm.

### 3.2. Interaction of AgNPs with Metal Ions

**Figure 2:**
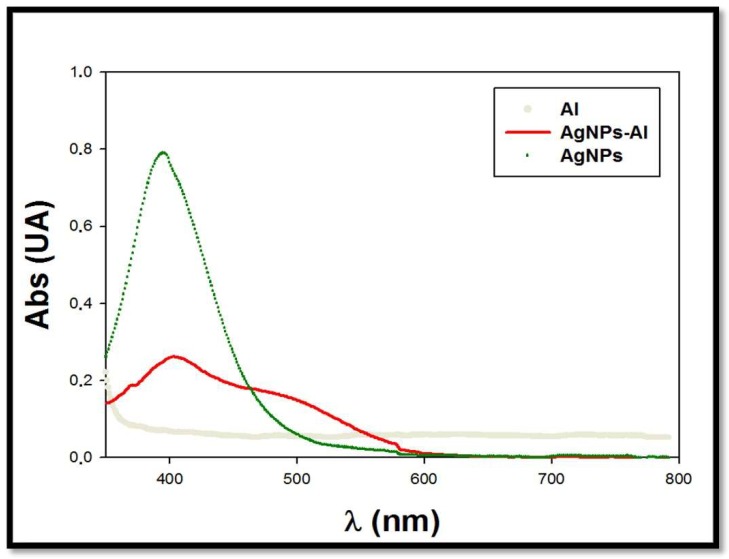
Spectra of the mixture citrate+PVP-AgNPs:Al. C_Al(III)_ = 100 mM; C_Citrate+PVP-AgNPs_ = 7.6 × 10^−15^ M.

Mixtures of the four kinds of synthesized AgNPs solutions (concentration ranging between 7.6 × 10^−15^ and 2.72 × 10^−11^ M) with different metal ions in solution (100 mM) were prepared to evaluate the possible selectivity of the AgNPs toward some of these metal ions. For the preliminary assays, Co(II), Cr(III), Cr(VI), Ni(II), Cu(II), Fe(III,) and Al(III) were selected as metallic ions. In each case, the solution pH was acidified with nitric acid to ensure the desired oxidation state of the metallic ions. Absorption spectra were measured for all the prepared mixtures. Only in the case of Al(III) was a possible interaction band observed in the spectra. This effect only occurred in the case of AgNPs stabilized with PVP, especially citrate+PVP-AgNPs. Figure 2 shows the obtained spectra for the mixture citrate+PVP-AgNPs (7.6 × 10^−15^ M) in the presence of a 100 mM Al(III) solution. In the figure, the spectra corresponding to a silver nanoparticles solution (7.6 × 10^−15^ M) and a 100 mM Al(III) solution are also plotted. 

The appearance of a band in the spectra around 500 nm can be observed, possibly due to the interaction between aluminum and AgNPs. We also observed a change in the color of the mixture, which was corroborated by the appearance of a blue shift in the corresponding absorption spectrum. When using a low concentration of silver nanoparticles (7.6 × 10^−15^ M), a good signal-to-noise ratio was obtained for the measured plasmon signal. Regarding a possible influence of the AgNPs concentration in the efficiency of the sensor, for lower AgNPs concentrations, discriminating between signal and noise in the system would be difficult. The concentration of nanoclusters used for the study could be slightly increased but this would have less of an effect on the plasmon shift by the analyte, which would result in a decrease in the obtained detection limit.

Therefore, the interaction between citrate+PVP-AgNPs and aluminum was further studied by analyzing the influence of pH and metal concentration in solution.

#### 3.2.1. Effect of Medium pH

**Figure 3:**
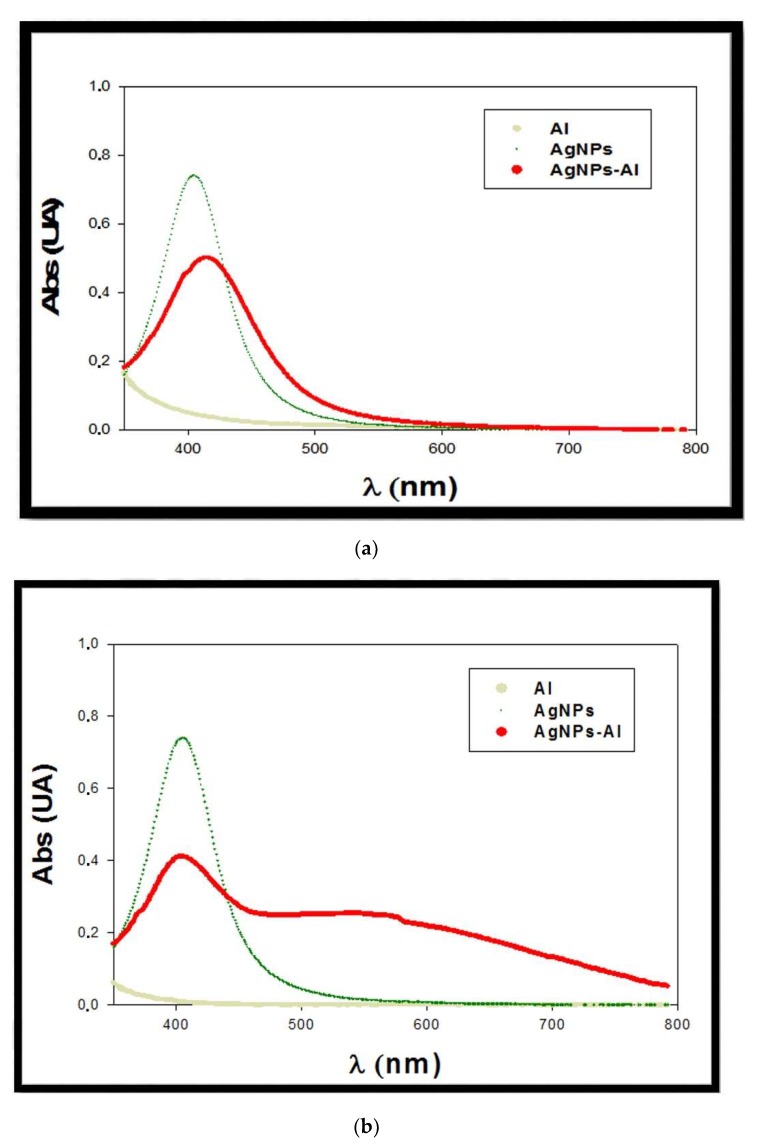
Influence of pH in the interaction of citrate+PVP-AgNPs:Al. (**a**) pH 3 and (**b**) pH 2. C_Al(III)_ = 100 mM; C_Citrate+PVP-AgNPs_ = 7.6 × 10^−15^ M.

Due to its importance as an experimental factor, the influence of solution pH in the interaction of aluminum ions with citrate+PVP-AgNPs was also studied. In this case, the studied pH ranged between 1 and 3 to assess the presence of Al(III) in solution. Different mixtures of citrate+PVP-AgNPs:Al were prepared adjusting the media pH at 1, 2 and 3, respectively, adding different amounts of nitric acid (5mM). The concentrations of citrate+PVP-AgNPs and Al(III) were 7.6 × 10^−15^ M and 100 mM, respectively. At pH 1, the characteristic light yellow color of the AgNPs solution immediately disappeared due to instability of the silver nanoparticles in the acidic media. This finding was checked when the spectrum was registered because the band centered at 400 nm disappeared, which corresponds to the free AgNPs in solution. Once registering the spectrum of the mixture citrate+PVP-AgNPs:Al at pH 3, the maximal absorption of the spectra drastically decreased. We observed a slight displacement toward higher wavelengths compared with the citrate+PVP-AgNPs spectrum (Figure 3a), and band broadening also appreciated. A possible reason for this behavior may be the appearance of Al(OH)_3_ in solution at this pH or even the possibility of ion pair formation [39]. The stability of the mixture was also checked by registering the absorption spectra every 15 min over 48 h. A decrease of 70% in the maximal absorption was found during the first 15 min, with the maximum absorption of the spectra almost disappearing after 24 h. 

Absorption spectra were registered at pH 2. Figure 3b reveals that under these conditions, the maximal absorption also decreased. Simultaneously, a new broad band (located between 470 and 750 nm) was found due to the interaction between citrate+PVP-AgNPs and the aluminum ions in the solution. The stability of the mixture at this pH was studied. In this case, spectra were registered during five days, observing a decrease of 50% in the maximal absorption after 24 h. From that time, registered spectra did not change for up to five days. This indicates a higher stability of the solutions at pH 2. Consequently, further experiments were conducted at pH 2 and within 24 h after preparing the citrate+PVP-AgNPs:Al mixtures. 

#### 3.2.2. Influence of Aluminum Concentration

**Figure 4:**
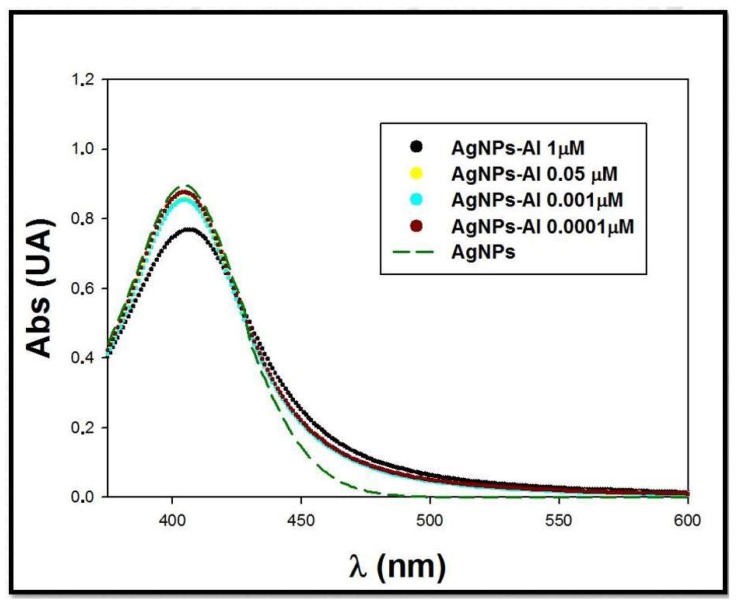
Spectra of mixtures citrate+PVP-AgNPs:Al. C_Citrate+PVP-AgNPs_ = 7.6 × 10^−15^ M. C_Al(III)_ = 1–1 × 10^−4^ μM.

To take advantage of the interaction between silver nanoparticles and aluminum as a possible sensor for determining the presence of this metallic ion in solution, the influence of Al(III) concentration was evaluated. Within this realm, although preliminary assays were completed using 100 mM Al(III) solutions, metallic ion concentration was decreased for further study. Therefore, mixtures containing a fixed amount of citrate+PVP-AgNPs (7.6 × 10^−15^ M) and decreasing amounts of Al(III), ranging between 1 μ and 1 × 10^−4^ μM, were prepared. The final pH of the mixtures was adjusted to pH 2 using an aqueous nitric acid solution (5 mM). The spectra of the prepared mixtures were registered and the results are shown in Figure 4. The spectra corresponding to a citrate+PVP-AgNPs solution in the same concentration as in the mixtures were acquired. We observed that an increase in Al(III) concentration in the mixtures induced a decrease in the citrate+PVP-AgNPs maximal absorption. A broadening of the band in the region of 460–580 nm occurred. This behavior is characteristic for an interaction process between silver nanoparticles and aluminum, even at low amounts of this metallic ion in solution.

### 3.3. Deconvolution of Spectra Citrate+PVP-AgNPs:Al 

After these experimental studies, the obtained results suggested the existence of a direct interaction between Al(III) and citrate+PVP-AgNPs that produces substantial changes in the experimental spectra. To further study this interaction, a deconvolution procedure was performed for surface plasmon resonance bands [40]. It is possible to split the experimental spectra to obtain the contribution of each individual band to the global spectra. In this case, there were four individual bands corresponding to: sample matrix (B1), free silver nanoparticles in solution (B2), silver nanoparticles interacting with Al(III) (B3), and aggregated silver nanoparticles (B4). The sum of these individual bands led to the global spectra (DEC), which should correspond to the experimental one (EXP). The characteristic parameters of each band were also determined using the deconvolution procedure, including the maximum absorbance (*A*_max_), the wavelength corresponding to the maximum absorption (*λ*_max_), and the half width of the band (*w_v_*).

The absorbance of each individual band can be calculated from the following expression:

(1) A=Amaxexp〈−ln2{ln[1+2(λmax−λ)λmax λksinh(b)wv]b}2〉

where *k* is a proportionality constant, *λ* is the radiation wavelength, and *b* is an index of the band asymmetry. In our case, spectral bands were approximated by Gaussian curves, so *b* in Equation (1) was set to a low value (i.e., *b* = 10^−4^) to ensure the symmetry of the analyzed bands. The parameter *w_v_* is defined as:

(2) $$w_{v}=\nu_{u}-\nu_{l}$$

where *ν_u_* and *ν_l_* are the upper and lower wavenumber values, respectively, in which *A* falls to 1/2 *A*_max_. Thus, with *k* = 10^4^, *ν* and *λ* are expressed in kilokaysers (1 kK = l0^3^ cm^−1^) and nanometers, respectively. The simulated spectra were calculated using Decouvis software [41], minimizing the global residual function (*δ_Fit_*), which is defined as:

(3) $$\delta_{Fit}=\sum_{i=1}^{n}{\left(A_{\exp}-A_{calc}\right)}^{2}$$

where *A_exp_* corresponds to the experimental absorbances and *A_calc_* to the absorbances calculated according to Equation (1).

**Figure 5:**
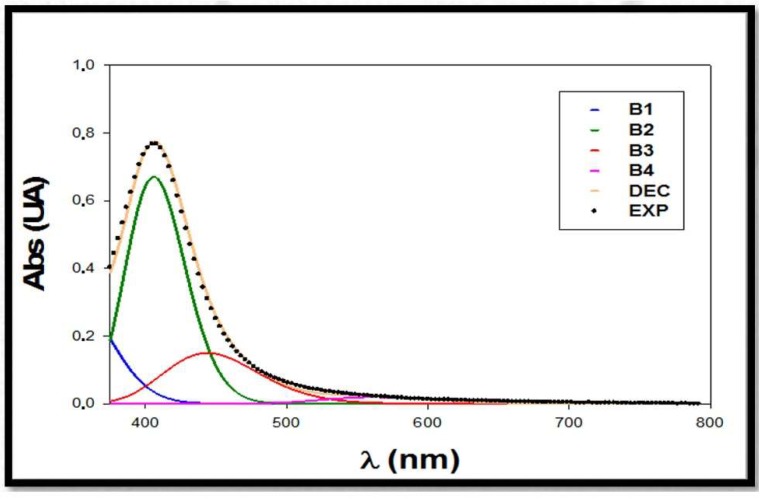
Deconvoluted spectra of a mixture citrate+PVP-AgNPs:Al, corresponding to an Al(III) concentration of 1 μM. EXP: experimental spectra; DEC: deconvoluted spectra; B1: sample matrix band; B2: free silver nanoparticles in solution band; B3: silver nanoparticles interacting with Al(III) band; B4: aggregated silver nanoparticles band.

To illustrate the deconvolution process, Figure 5 shows a deconvoluted spectra of a mixture citrate+PVP-AgNPs:Al, corresponding to an Al(III) concentration of 1 μM. The individual bands B1, B2, B3, and B4 can be seen as well as their sum, which is the deconvoluted spectra (DEC). The experimental spectra of the mixture is also plotted. A good agreement was obtained between experimental and theoretical spectra, with the B3 band being sensitive to the aluminum content in solution. 

Thus, the deconvolution procedure was applied to all of the citrate+PVP*-*AgNPs:Al experimental spectra for each Al(III) concentration investigated, and the band characteristic parameters (*A*_max_, *λ*_max_ and *w_v_*) were calculated for each measured spectrum. The obtained parameters corresponding to the sample matrix ($A_{\max}^{B1}$ = 0.330 U.A., *λ*_max_ = 355 nm, and *w_v_* = 4.1 kK) and aggregated silver nanoparticles ($A_{\max}^{B4}$ = 0.020 U.A., *λ*_max_ = 561 nm, and *w_v_* = 2.9) were kept constant for all the mixtures because both bands showed the same contribution in all the analyzed solutions. The parameters obtained for the free silver nanoparticles in solution B2 and silver nanoparticles interacting with Al(III) B3 are listed in Table 1. We noted a clear tendency in the absorbance values, which were proportional to the species concentration in solution. Thus, as the Al(III) concentration increases, the value of $A_{\max}^{B3}$ is amplified, while the $A_{\max}^{B2}$ diminishes, corroborating the direct interaction process. This effect is demonstrated through the $A_{\max}^{B3}$/$A_{\max}^{B2}$ ratio in the Table 2. The maximum absorption wavelength for B2 ($\lambda_{\max}^{B2}$ = 406 nm) and B3 ($\lambda_{\max}^{B3}$ = 444 nm) bands remained constant, which is consistent with the nature of the interaction process.

Once the experimental spectra were deconvoluted and the individual contribution of each species present in solution was known, the *A_max_* parameter for free AgNPs ($A_{\max}^{B2}$) and interacting AgNPs ($A_{\max}^{B3}$) can offer valuable information through the calculation of the relative maximum absorbance ratio $A_{\max}^{B3}$/$A_{\max}^{B2}$. 

### 3.4. Analytical Study of the Citrate+PVP-AgNPs:Al Interaction

**Figure 6:**
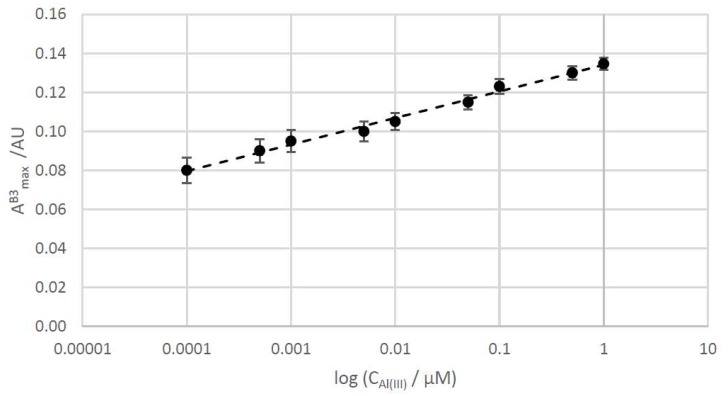
Calibration line of the $A_{\max}^{B3}$ versus different concentrations of aluminum.

The interaction between silver nanoparticles and Al(III) was also studied from an analytical point of view. For sensing purposes, the interaction band between citrate+PVP-AgNPs and aluminum $(A_{\max}^{B3}$) can be used as analytical signal for determining very low amounts of this metal in solution. Therefore, analytical quality parameters, such as linearity, sensitivity, and precision, were evaluated. In the first step, linearity was checked by plotting $A_{\max}^{B3}$ versus concentration of Al(III) in a range of 10^−4^ μM to 1 μM. The calibration equation was $A_{\max}^{B3}=0.0148\;logC_{Al\left(III\right)}+0.1472$. Good linearity was obtained (98.0%), calculated as 100(1 − S_b_/b), where b is the slope of the regression line and S_b_ is its standard deviation. Figure 6 shows the obtained calibration line. Due to the wide range of concentrations, a logarithmic scale was chosen to visualize the regression line. 

Limits of detection (LOD) and limit of quantitation (LOQ) were estimated to evaluate the sensitivity of silver nanoparticles to detect low amounts of metal in solution. LOD and LOQ were calculated as the concentration of Al(III), producing a signal (maximal absorbance) equal to 3 and 10 times the standard deviation of the blank signal, respectively [42]. In this case, maximal absorbance, corresponding to B1 ($A_{\max}^{B1}$) in the spectra of the mixture containing lower amounts of Al(III) (10^−4^ μM), was considered the blank signal [43]. The obtained values for LOD and LOQ were 4.05 × 10^−2^ and 13.5·× 10^−2^ μM Al(III), respectively. These values indicate that citrate+PVP-AgNPs can be used as sensor for detecting the presence of Al(III) in solution with high sensitivity. 

To evaluate the precision of the proposed aluminum detection system, repeatability and intermediate precision were evaluated. Mixtures of citrate+PVP*-*AgNPs:Al were prepared containing Al(III) amounts at three levels of concentrations, ranging between 10^−4^ and 1 μM. Single day measurements (repeatability) were recorded, registering spectra of the system every hour. Weekly measurements (three days per week) were also recorded for calculating intermediate precision. Once spectra were deconvoluted, repeatability and intermediate precision were estimated as relative standard deviation (%RSD) of the corresponding $A_{\max}^{B3}$ data, obtaining %RSD_repeatability_ < 8% and %RSD_int.precision_ < 12%, respectively. 

**Figure 7:**
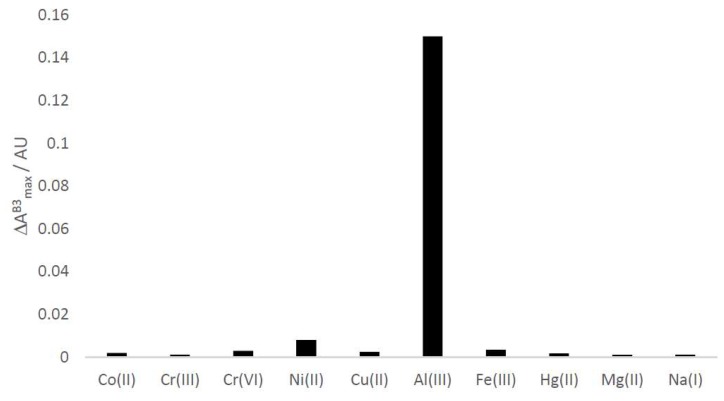
The variation in the maximum absorbance on the interaction band ($A_{\max}^{B3})$ in the presence of different metal ions.

Selectivity toward aluminum over other competitive metal ions was also studied. Several metal ions, such as Co(II), Cr(III), Cr(VI), Ni(II), Cu(II), Fe(III), Hg(II), Mg(II), and Na(I) were tested under optimized conditions of the proposed citrate+PVP-AgNPs-based sensor. None of the tested ions produced an appreciable change in the measured spectra at the same wavelength where citrate+PVP-AgNPs interact with Al(III) ions ($A_{\max}^{B3})$, as shown in Figure 7. Only in the presence of Ni(II) was a slight increase of 5% of the analytical signal detected when the concentration ratio Ni(II)/Al(III) was 100:1. Therefore, we verified the high selectivity of the proposed sensor for aluminum ions. 

Consequently, after the validation study, we determined that the proposed aluminum detection system based on AgNPs is suitable according to the Association of Official Analytical Chemists (AOAC) guide [44].

Comparing the obtained LOD and LOQ values for the proposed method with those reported by other authors who used different AgNPs/AuNPs-based sensors for detecting aluminum ions (Table 3) [4,29,45,46,47,48], we found that the citrate+PVP*-*AgNPs-based sensoring system provides lower limits of detection and quantitation. Only a sensor using AgNPs capped with 8-hydroxyquinoline-5-sulfonate offers lower LOD. 

Therefore, the proposed sensor based on citrate+PVP-AgNPs seems to be a good alternative for the determination of aluminum ions in solution.

### 3.5. Real Water Samples Analysis 

**Figure 8:**
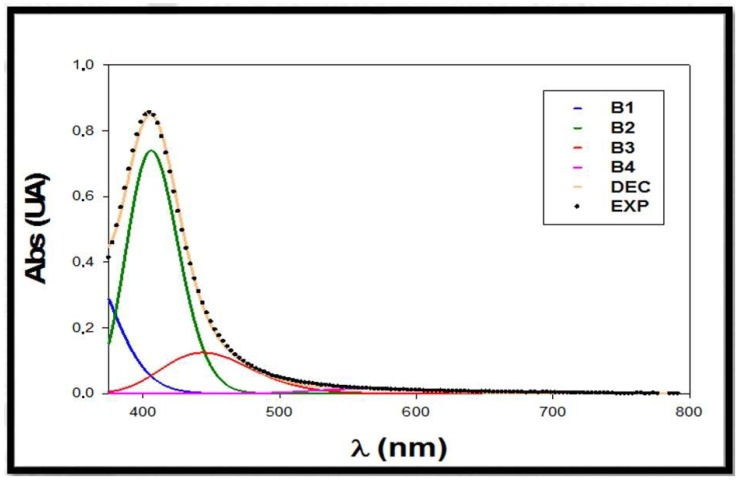
Deconvoluted spectra of a mixture citrate+PVP-AgNPs:mineral water sample. EXP: experimental spectra; DEC: deconvoluted spectra; B1: sample matrix band; B2: free silver nanoparticles in solution band; B3: silver nanoparticles interacting with Al(III) band in mineral water sample; and B4: aggregated silver nanoparticles band.

Citrate+PVP-AgNPs were applied as a sensor for the detection of aluminum in real water samples using the proposed sensing strategy in tap water, pond water, river water, and mineral water. For this purpose, several samples of tap water and pond water were collected in the urban area of Seville (Spain). River water samples were collected from the Guadalquivir River and mineral water samples were acquired in commercial stores. All samples were filtered through disposable nylon filters (0.2 μm) before analysis. We mixed 300 μL of water sample with 1200 μL of citrate+PVP-AgNPs solution, an adequate amount of Al(III) standard solution (0–1 μM) and nitric acid (5 mM) for adjusting pH media to 2, with final volume of the mixture being 2 mL. The corresponding spectra were registered and the described deconvolution process was applied. Figure 8 shows the deconvoluted spectrum of the mixture citrate+PVP*-*AgNPs:mineral water. 

The concentration of Al(III) for each water sample was calculated from the regression line, calculating the average recovery according to the standard addition method. The obtained data are summarized in Table 4. For comparison purposes, data obtained by ICP-MS for the water samples are also included.

The recovery of the analyzed samples ranged between 96% and 113%, showing that *+*PVP-AgNPs can be considered a good detection system for Al(III) in real water samples. 

As abovementioned, data about Al(III) detection using functionalized silver nanoparticles were reported in the literature. The obtained LOD value for the proposed detection system in this work is two orders lower than those reported in the literature [18,28,45,46]. AgNPs-based detection systems were also applied to water samples, obtaining similar results to those provided by the proposed method. Within this realm, functionalized gold nanoparticles (AuNPs) have also been used for the selective detection of Al(III) [26,27,47]. The obtained LOD values in these studies are of the same order as the LOD of the proposed system. Thus, the citrate+PVP-AgNPs-based detection system for Al(III) in this work seems to be a good and easier alternative for detecting this metal in solution.

## 4. Conclusions

This paper proposes an AgNPs-based system for the selective detection of Al(III). Silver nanoparticles stabilized with citrate and polyvinylpyrrolidone were used as an aluminum sensor. To the best of our knowledge, this is the first time that non-functionalized silver nanoparticles were used for the selective detection of this metallic ion in solution. A deconvolution process was applied to identify the interaction band between citrate+PVP-AgNPs and Al(III), with the maximal absorbance of this band being the analytical signal used for detecting the presence of aluminum in solution. 

Linearity, sensitivity (LOD and LOQ), and precision of the proposed aluminum detection system were studied. Good linearity was found between $A_{\max}^{B3})$ and Al(III) concentration (98.0%). High sensitivity (LOD = 4.05 × 10^−2^ μM; LOQ = 13.5 × 10^−2^ μM) and good repeatability (%RSD < 8%) as well as intermediate precision (%RSD < 12%) were obtained. Citrate+PVP-AgNPs were satisfactorily used for detecting Al(III) in real water samples.

## Figures and Tables

**Table 1:** Characterization data of the synthetized silver nanoparticles.

Silver Nanoparticle	Shape	Medium Size (nm)	λ_max_ (nm)
PVP-AgNPs	spherical	10–20	424
NaBH_4_-AgNPs	spherical	2–4	392
Citrate-AgNPs	spherical	12–26 30–60	424
Citrate+PVP-AgNPs	spherical	10–20 25–45	397

**Table 2:** Deconvolution parameters of the bands corresponding to free silver nanoparticles in solution (B2) and silver nanoparticles interacting with Al(III) (B3). $\lambda_{\max}^{B2}$ = 406 nm; $\lambda_{\max}^{B3}$ = 444 nm.

Al(III) (μM) in Mixtures	B2		B3		$A_{max}^{B3}$ $/A_{max}^{B2}$
	*A*_max_ (U.A.)	*w_v_* (kK)	*A*_max_ (U.A.)	*w_v_* (kK)	
1	0.670	3.0	0.150	4.0	0.22
0.5	0.690	2.7	0.140	3.9	0.20
0.1	0.700	2.7	0.135	3.9	0.19
5 × 10^−2^	0.740	2.7	0.125	3.9	0.17
1 × 10^−2^	0.753	2.7	0.120	3.9	0.16
5 × 10^−3^	0.760	2.7	0.110	3.9	0.14
1 × 10^−3^	0.765	2.9	0.105	4.0	0.13
5 × 10^−4^	0.770	2.9	0.095	4.4	0.12
1 × 10^−4^	0.775	3.1	0.090	4.4	0.11

**Table 3:** Comparison of the sensitivity data using different AgNPs/AuNPs-based sensors.

Probe	10^2^ LOD (μM)	10^2^ LOQ (μM)	Reference
Citrate+PVP*-*AgNPs	4.05	13.5	This work
Alizarin red S-AgNPs	12	40.0	[4]
Silver-gold alloy nanoclusters	80	266.4	[45]
Silver nanoclusters	10	33.3	[30]
Schiff base-functionalized AuNPs	29	96.6	[46]
Surface functionalized AuNPs	6.7	22.3	[47]
8-hydroxyquinoline-5-sulfonate-AgNPs	0.2	0.666	[48]

**Table 4:** Determination of Al(III) in real water samples.

Sample	Al(III) Added (μM)	Al(III) Observed (μM) * Citrate+PVP-AgNPs	Recovery (%)	Al(III) Observed (μM) * ICP-MS
Tap water	0	0.23 ± 0.05	-	0.22 ± 0.03
	0.5	0.82 ± 0.04	112	0.79 ± 0.02
	1	1.3 ± 0.1	106	1.4 ± 0.2
Pond water	0	0.4 ± 0.1	-	0.3 ± 0.2
	0.5	1.02 ± 0.08	113	1.06 ± 0.06
	1	1.6 ± 0.2	107	1.4 ± 0.3
River water	0	0.56 ± 0.08	-	0.60 ± 0.03
	0.5	1.05 ± 0.05	99	1.02 ± 0.06
	1	1.5 ± 0.1	96	1.7 ± 0.1
Mineral water	0	0.16 ± 0.07	-	0.14 ± 0.04
	0.5	0.8 ± 0.2	106	0.7 ± 0.2
	1	0.14 ± 0.03	98	0.16 ± 0.05

* The average of triplicate determinations.

## References

[1] Baylor W., Egan W., Richman P. (2002). Aluminum salts in vaccines-US perspective. Vaccine.

[2] Soni M.G., White S.M., Flamm W.G., Burdock G.A. (2001). Safety evaluation of dietary aluminum. Regul. Toxicol. Pharm..

[3] Lee J., Kim H., Kim S., Noh J.Y., Song E.J., Kim C., Kim J. (2013). Fluorescent dye containing phenol-pyridyl for selective detection of aluminum ions. Dye. Pigment..

[4] Liu X., Wu F., Ma L. (2014). Colorimetric assay for Al^3+^ based on alizarin red S-functionalized silver nanoparticles. Aust. J. Chem..

[5] Banks W.A., Kastin A.J. (1989). Aluminum-induced neurotoxicity: Alterations in membrane function at the blood-brain barrier. Neurosci. Biobehav. Rev..

[6] Good P.F., Olanow C.W., Perl D.P. (1992). Neuromelanin-containing neurons of the substantia nigra accumulate iron and aluminum in parkinson’s disease: A LAMMA study. Brain Res..

[7] Paik S.R., Lee J.H., Kim D.H., Chang C.S., Kim J. (1997). Aluminum-induced structural alterations of the precursor of the non-Aβ component of alzheimer’s disease amyloid. Arch. Biochem. Biophys..

[8] Lin J.L., Kou M.T., Leu M.L. (1996). Effect of long-term low-dose aluminum-containing agents on hemoglobin synthesis in patients with chronic renal insufficiency. Nephron.

[9] ATSDR (1992). Toxicological Profile for Aluminium.

[10] WHO (1998). Health criteria and other supporting information. Guidelines for Drinking-Water Quality.

[11] Kaur A., Raj T., Kaur S., Kaur N. (2014). Nano molar detection of Al^3+^ in aqueous medium and acidic soil using chromone based fluorescent organic nanoparticles (FONPs). Anal. Methods.

[12] Gupta V.K., Singh A.K., Kumawat L.K. (2014). Thiazole Schiff base turn-on fluorescent chemosensor for Al^3+^ ion. Sens. Actuators B.

[13] Deschaume O., Fournier A., Shafranab K.L., Perry C.C. (2008). Interactions of aluminium hydrolytic species with biomolecules. New J. Chem..

[14] Gupta V.K., Goyal R.N., Jain A.K., Sharma R.A. (2009). Aluminium (III)-selective PVC membrane sensor based on a schiff base complex of N,N’-bis (salicylidene)-1,2-cyclohexanediamine. Electrochim. Acta.

[15] Shervedani R.K., Rezvaninia Z., Sabzyan H., Boeini H.Z. (2014). Characterization of gold-thiol-8-hydroxyquinoline self-assembled monolayers for selective recognition of aluminum ion using voltammetry and electrochemical impedance spectroscopy. Anal. Chim. Acta.

[16] Satiroglu N., Tokgoz I. (2010). Cloud point extraction of aluminum (III) in water samples and determination by electrothermal atomic absorption spectrometry, flame atomic absorption spectrometry and UV-visible spectrophotometry. Int. J. Environ. Anal. Chem..

[17] Nagaoka M.H., Maitani T. (2009). Speciation of Aluminium in Human Serum Investigated by HPLC/High Resolution Inductively Coupled Plasma Mass Spectrometry (HR-ICP-MS): Effects of Sialic Acid Residues of the Carbohydrate Chain on the Binding Affinity of Aluminium for Transferrin. J. Health Sci..

[18] Chen S., Fang Y.M., Xiao Q., Li J., Li S.B., Chen H.J., Sun J.J., Yang H.H. (2012). Rapid visual detection of aluminium ion using citrate capped gold nanoparticle. Analyst.

[19] Gupta V.K., Singh A.K., Ganjali M.R., Norouzi P., Faridbod F., Mergu N. (2013). Comparative study of colorimetric sensors based on newly synthesized schiff bases. Sens. Actuators B Chem..

[20] Sung H.K., Oh S.Y., Park C., Kim Y. (2013). Colorimetric detection of Co^2+^ ion using silver nanoparticles with spherical, plate, and rod shapes. Langmuir.

[21] Shang Y., Wu F., Qi L. (2012). Highly selective colorimetric assay for nickel ion using N-acetyl-L-cysteine-functionalized silver nanoparticles. J. Nanopart. Res..

[22] Bothra S., Solanki J.N., Sahoo S.K. (2013). Functionalized silver nanoparticles as chemosensor for pH, Hg^2+^ and Fe^3+^ in aqueous medium. Sens. Actuators B.

[23] Kumar V.V., Anbarasan S., Christena L.R., SaiSubramanian N., Anthony S.P. (2014). Bio-functionalized silver nanoparticles for selective colorimetric sensing of toxic metal ions and antimicrobial studies. Spectrochim. Acta Part A Mol. Biomol. Spectrosc..

[24] Gao Y.X., Xin J.W., Shen Z.Y., Pan W., Li X., Wu A.G. (2013). A new rapid colorimetric detection method of Mn^2+^ based on tripolyphospfate modified silver nanoparticles. Sens. Actuators B.

[25] Chen Z., Huang Y., Li X., Zhou T., Ma H., Qiang H., Liu Y. (2013). Colorimetric detection of potassium ions using aptamer-functionalized gold nanoparticles. Anal. Chim. Acta.

[26] Amanulla B., Perumal K.N., Ramaraj S.K. (2019). Chitosan functionalized gold nanoparticles assembled on sulphur doped graphitic carbón nitride as a new platform for colorimetric detection of trace Hg^2+^. Sens. Actuators B Chem..

[27] Chen Y., Lee I., Sung Y., Wu S. (2013). Colorimetric detection of Al^3+^ using triazole-ether functionalized gold nanoparticles. Talanta.

[28] Rastogi L., Dash K., Ballal A. (2017). Selective colorimetric/visual detection of Al^3+^ in ground water using ascorbic acid capped gold nanoparticles. Sens. Actuators B Chem..

[29] Yang N., Gao Y., Zhang Y., Shen Z., Wu A. (2014). A new rapid colorimetric detection method of Al^3+^ with high sensitivity and excellent selectivity based on new mechanism of aggregation of smaller etched silver nanoparticles. Talanta.

[30] Liu X., Shao C., Chen T., He Z., Du G. (2019). Stable silver nanoclusters with aggregation-induced emission enhancement for detection of aluminum ion. Sens. Actuators B Chem..

[31] Perinot A., Kshirsagar P., Malvindi M.A., Pompa P.P., Fiammengo R., Caironi M. (2016). Direct-written polymer field-effect transistors operating at 20 MHz. Sci. Rep..

[32] Kshirsagar P., Sangaru S.S., Brunetti V., Malvindi M.A., Pompa P.P. (2014). Synthesis of fluorescent metal nanoparticles in aqueous solution by photochemical reduction. Nanotechnology.

[33] Zhang Z., Zhang H. (2015). Controllable synthesis of silver nanoparticles in hyperbranched macromolecule templates for printed flexible electronics. RSC Adv..

[34] Prado-Gotor R., López-Pérez G., Martín M.J., Cabrera-Escribano F., Franconetti A. (2014). Use of gold nanoparticles as crosslink agent to form chitosan nanocapsules: Study of the direct interaction in aqueous solutions. J. Inorg. Bichem..

[35] López-Pérez G., Prado-Gotor R., Fuentes-Rojas J.A., Martin-Valero M.J. (2020). Understanding gold nanoparticles interactions with chitosan: Crosslinking agents as novel strategy for direct covalent immobilization of biomolecules on metallic surfaces. J. Mol. Liq..

[36] Wang H.S., Qiao X.L., Chen J.G., Wang X.J., Ding S.Y. (2005). Mechanism of PVP in the preparation of silver nanoparticles. Mater. Chem. Phys..

[37] Van Dong P., Ha C.H., Binh L.T., Kasbohm J. (2012). Chemical synthesis and antibacterial activity of novel-shaped silver nanoparticles. Int. Nano Lett..

[38] Fang J., Zhong C., Mu R. (2005). The study of deposited silver particulate films by simple method for efficient SERS. Chem. Phys. Lett..

[39] Molero-Casado M., González-Arjona D., Calvente-Pacheco J.J., López-Pérez G. (1999). Activity Coefficients of Al(ClO_4_)_3_ in Aqueous Solutions: A Reexamination. J. Electroanal. Chem..

[40] Sevilla J.M., Dominguez M., García-Blanco F., Blázquez M. (1989). Reolution of absorption spectra. Comput. Chem..

[41] González-Arjona D. (1995). DECOUVIS.

[42] Cuadros L., García A.M., Bosque J.M. (1996). Statistical estimation of linear calibration range. Anal. Lett..

[43] Compañó I., Beltrán R., Ríos Castro A. (2002). Garantía de Calidad en los Laboratorios Analíticos.

[44] (2013). AOAC Official Methods Program Manual, Part 6: Guidelines for Collaborative Study. http://www.aoac.org/.

[45] Zhou T., Lin L., Rong M., Jiang Y., Chen X. (2013). Silver-gold alloy nanoclusters as a fluorescence-enhanced probe for aluminum ion sensing. Anal. Chem..

[46] Huang P., Li J., Liu X., Wu F. (2016). Colorimetric determination of aluminum (III) based on the aggregation of Schiff base-functionalized gold nanoparticles. Microchim. Acta.

[47] Shinde S., Kim D.-Y., Saratale R.G., Syed A., Ameen F., Ghodake G. (2017). A spectral probe for detection of aluminum (III) ions using Surface functionalized gold nanoparticles. Nanomaterials.

[48] Shang Y., Gao D., Wu F., Wan X. (2013). Silver nanoparticles capped with 8-hydroxyquinoline-5-sulfonate for the determination of trace aluminum in water samples and for intracellular fluorescence imaging. Microchim. Acta.

